# Trends in cataract surgical treatment within the Brazilian national public health system over a 20-year period: Implications for Universal Eye Health as a global public health goal

**DOI:** 10.1371/journal.pgph.0000328

**Published:** 2022-06-09

**Authors:** Arthur Gustavo Fernandes, Aline Nunes Ferraz, Rafael da Silva Lemos, Sung Eun Song Watanabe, Adriana Berezovsky, Solange Rios Salomão

**Affiliations:** 1 Department of Ophthalmology and Visual Sciences, Paulista Medical School, Federal University of São Paulo – UNIFESP, São Paulo, São Paulo, Brazil; 2 Department of Anthropology and Archaeology, University of Calgary, Calgary, Alberta, Canada; University of Birmingham, UNITED KINGDOM

## Abstract

Cataract is a highly prevalent, treatable, and sight threatening condition considered one of the main focuses of public health policies addressing visual impairment and blindness towards Universal Eye Health. We aimed to investigate the trends on number of cataract surgical procedures performed through the Brazilian national health system (SUS) from 2000 to 2019 while also evaluating costs associated with it. The Brazilian Public Health System Information Database (DATASUS) was used as the primary data source for procedures including extracapsular cataract extraction (ECCE) and phacoemulsification. Trends along the years were evaluated through generalized linear models. A total of 8,424,521 cataract procedures were performed from 2000 to 2019, with a significant increase along the years from 228,145 in 2000 to 663,186 in 2019 (p<0.001), a cataract surgical procedure rate change from 13.15 to 32.28 procedures per 10,000 people. It was observed a significant increase on the number of phacoemulsification (p<0.001) and a significant decrease on the number of ECCE (p<0.001). A shift on the predominant technique has occurred between 2007 and 2008 with phacoemulsification increasing its percentual representativity from 34.3% to 69.7% of all procedures, reaching 96.1% in 2019. Phacoemulsification costs per procedure increased 30.5% from from USD$119.00 to USD$155.33 (p = 0.007) and the ECCE costs per procedure increased 29.1% from USD$78.57 to USD$101.43 (p = 0.001). There is an increasing trend of procedures related to cataract treatment performed through SUS along the 20-years period and a switch on the technique predominance from ECCE to phacoemulsification was observed after 2007. The costs associated with both techniques have increased but have not followed the country’s overall inflation. Data derived from DATASUS is important to understand the overall panorama of ocular health offered by the national health system and to provide information to guide healthcare leaders on management and planning of public health policies within the system.

## Introduction

Universal Eye Health (UEH) is the main goal from the World Health Organization (WHO) action plan launched in 2014 aiming to reduce avoidable visual impairment as a global public health issue and to secure access to rehabilitation services for the visually impaired [1, 2]. In general, the UEH action plan redefined the previous Vision 2020 program aiming to offer comprehensive eye care services including eye health promotion, prevention, treatment, and rehabilitation; to integrate eye health into the wider health system; to provide access for everyone, including the poor, minorities, and the disabled; and to ensure that payment for services does not prevent access [1–3].

Cataract is a condition characterized by the natural intraocular lens opacification. The lens is a structure derived from the ectodermal tissue and it is formed by epithelial cells that constantly generates lens fibers throughout life [4]. Differently than what occurs in the skin tissue, old lens fibers are not loss and so as result of the aging process, the lens become more compact and thicker, leading to loss on its transparency and to the cataract formation [4–6]. Although aging is pointed as the main risk factor for cataract, its development is a multifactorial process including personal and environmental risk factors as ethnicity, genotype, smoking status, ultraviolet exposition, diabetes, among other [4, 6, 7]. Different risk factors may lead to specific types of cataracts [4]. According to the most recent estimates, globally, there are 43.3 million people blind and other 295.3 million people moderate to severe visually impaired [8]. Cataract is reported as the main cause of blindness (15.2 million cases) and the second main cause of moderate to severe visual impairment (78.8 million cases) in adults aged 50 years or more [9]. This is a reversible condition and therefore one of the main focuses of public health policies addressing visual impairment and blindness worldwide. It has been recognized the need to increase the cataract surgical rate (annual number of cataract operations performed per million population) mainly in developing countries with extensive territory and regional discrepancies [10, 11].

Cataract treatment is surgical and it is only indicated when the patient refers substantial visual function decrease. Since the 800 BC cataract surgery was already performed using the couching technique of dislocate the lens from the visual axis by using needles [4]. By the mid-18^th^ century, the intracapsular cataract extraction (ICCE) was developed as a technique of removal of the entire lens including its capsule by an incision in the limbus [4, 12]. After the intraocular lens (IOL) development in the mid-20^th^ century, the technique was refined into the extracapsular cataract extraction (ECCE) with a careful removal of the anterior capsule, removal from the lens nucleus and aspiration of the remaining material in order to leave an intact posterior lens capsule with the equatorial zonular attachments facilitating the IOL implant [12, 13]. Phacoemulsification was developed in 1967 and is currently the technique of choice for cataract treatment [12–14]. The main difference between ECCE and phacoemulsification is that, instead of removing the nucleus lens through a large incision, in the phacoemulsification technique a high-frequency ultrasonic probe is inserted through a 3mm corneal incision, delivers energy to emulsify the lens nucleus inside the eye and then the same probe aspires the fragments. The smaller the incision the better the outcomes are expected including better refractive status, less complication rates, and shorter surgical time [4, 12].

Brazil is one of the few countries worldwide that offers universal, free of charge health insurance financed by the central government (Sistema Único de Saúde—SUS) that provides medical attention to the entire Brazilian population in every medical specialty including Ophthalmology, from the most basic health attention to the most complex procedures provided in tertiary hospitals. The purpose of this study is to investigate the trends on the absolute number of cataract surgical procedures and their respective techniques performed through the Brazilian National Health Insurance (SUS) from 2000 to 2019 while also evaluating the costs associated with it.

## Materials and methods

Brazilian Public Health System Information Database (DATASUS) was used as the primary data source for the current study. DATASUS represents the primary effort of Brazilian Federal Government to collect data from the national health system and includes information from all public health hospitals throughout the country [15]. This database, originally intended for administrative purposes, contains data on all hospitalizations and procedures covered by SUS in Brazil. The version available online shows no patient’s personal identification and gather data on specific procedure codes, general demographic information, place and date of admission, and procedure costs [16]. The cost data available reflects the procedure value for the specific year and has no adjustment for inflation or any other manipulation. This is an open-access platform.

All SUS cataract procedures including ECCE and phacoemulsification with or without IOL implantation performed from 2000 to 2019 were selected for the current study, including those performed in hospital or ambulatorial facilities. Data from 2008 to 2019 were derived from the system considering the following codes: 405050097 and 405050100 for ECCE and 405050119 and 405050372 for phacoemulsification. Data from 2000 to 2007 were derived from the system considering the following codes: 0814507, 0814603, 0814614, 0814615 and 0814617 for ECCE and 0814616 and 0814618 for phacoemulsification. Number of procedures and costs were analyzed according to year and region.

Statistical analyses were performed using Stata/SE Statistical Software, Release 14.0, 2015 (Stata Corp, College Station, Texas, USA). Frequency tables were used for descriptive analysis. Trends along the years were evaluated through univariate generalized linear models. P values ≤.05 were considered statistically significant. The rates of procedures per population were calculated using the Census data available at DataSUS as denominators [16].

## Results

**Fig 1:**
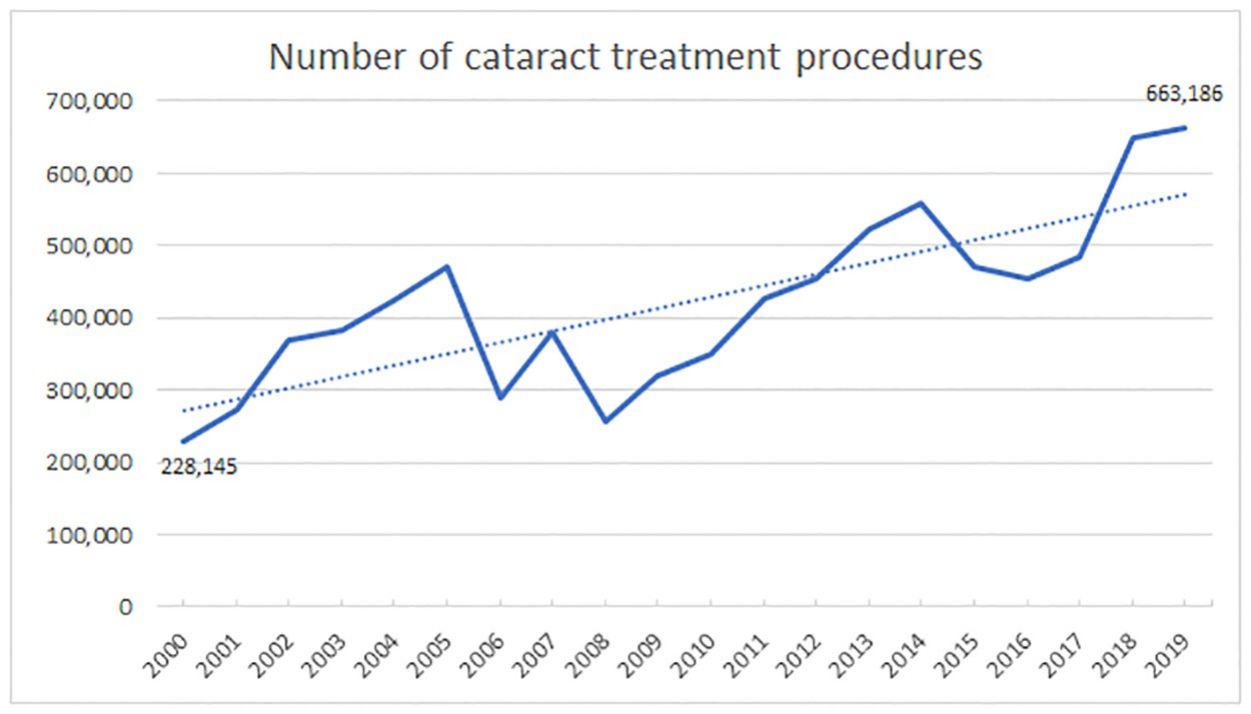
Trends in the absolute number of cataract treatment procedure from 2000 to 2019.

Along the 20-years period from 2000 to 2019, a total of 8,424,521 cataract treatment procedures were performed through the Brazilian National Health Insurance (SUS). Fig 1 shows the trends in the absolute number of cataract treatment procedure along the years.

Trend analysis shows a statistically significant increase on the number of procedures along the years (Coefficient: 17,149; 95% Confidence Interval [CI]: 12,070 to 22,227; p<0.001). It is observed an increase of 190.7% when comparing 2019 to 2000 (p<0.001). Table 1 shows the number of procedures according to the region where the procedure was carried out.

**Table 1:** Number of cataract treatment procedures according to the region.

Year	North	Northeast	Southeast	South	Midwest	All
**2000**	12,734	88,691	88,821	25,173	12,726	228,145
**2001**	18,941	103,739	104,898	25,955	20,646	274,179
**2002**	26,769	145,873	141,347	31,826	22,933	368,748
**2003**	20,025	162,942	141,645	35,935	23,307	383,854
**2004**	25,368	174,322	159,142	38,625	27,494	424,951
**2005**	32,824	197,099	168,408	44,569	26,662	469,562
**2006**	25,663	94,560	108,044	42,688	17,564	288,519
**2007**	32,780	116,120	152,982	53,638	24,984	380,504
**2008**	20,293	78,548	101,588	32,520	22,171	255,120
**2009**	20,977	118,510	112,838	42,926	25,168	320,419
**2010**	26,330	139,668	121,158	39,697	21,570	348,423
**2011**	54,062	146,238	157,637	43,204	25,429	426,570
**2012**	45,194	165,227	167,759	48,705	27,699	454,584
**2013**	52,583	174,413	211,947	56,865	27,808	523,616
**2014**	33,923	168,997	223,953	69,272	61,426	557,571
**2015**	29,468	122,844	206,469	72,307	39,158	470,246
**2016**	29,416	129,630	179,187	69,156	45,504	452,893
**2017**	31,123	128,181	204,681	77,250	42,295	483,530
**2018**	34,844	179,843	274,997	109,022	51,195	649,901
**2019**	33,626	201,179	277,246	119,858	31,277	663,186

When analyzing each region separately, increases of 376.1%, 212.1%, 164.1%, 145.8%, and 126.8% were observed for the South, Southeast, North, Midwest, and Northeast, respectively. Table 2 present the rate of cataract surgical procedures per total population in the different regions along the analyzed period.

**Table 2:** Rates of cataract surgical procedures per 10,000 people according to region.

Year	North	Northeast	Southeast	South	Midwest	All
**2000**	9.60	18.12	12.01	9.92	10.72	13.15
**2001**	13.95	20.93	14.00	10.10	17.04	15.59
**2002**	19.28	29.07	18.63	12.25	18.55	20.68
**2003**	14.11	32.08	18.45	13.67	18.49	21.25
**2004**	17.50	33.93	20.50	14.54	21.40	23.23
**2005**	22.19	37.94	21.46	16.61	20.37	25.36
**2006**	17.01	18.01	13.62	15.75	13.17	15.40
**2007**	21.32	21.90	19.09	19.61	18.40	20.08
**2008**	15.94	17.82	17.09	14.35	21.98	17.16
**2009**	15.52	24.72	17.12	17.74	24.38	19.73
**2010**	18.21	27.67	17.67	15.79	20.85	20.46
**2011**	34.68	28.42	21.79	16.56	23.01	24.05
**2012**	28.50	31.10	22.34	18.21	22.29	24.70
**2013**	32.92	32.14	26.27	20.57	20.11	27.18
**2014**	21.23	30.77	27.50	24.64	41.57	28.52
**2015**	18.27	22.27	25.02	25.29	26.03	23.80
**2016**	17.98	23.27	21.74	23.96	29.52	22.75
**2017**	18.72	22.79	24.53	26.50	26.93	24.01
**2018**	20.62	31.91	32.51	37.03	32.17	31.93
**2019**	19.78	35.47	32.38	40.30	19.66	32.28

**Fig 2:**
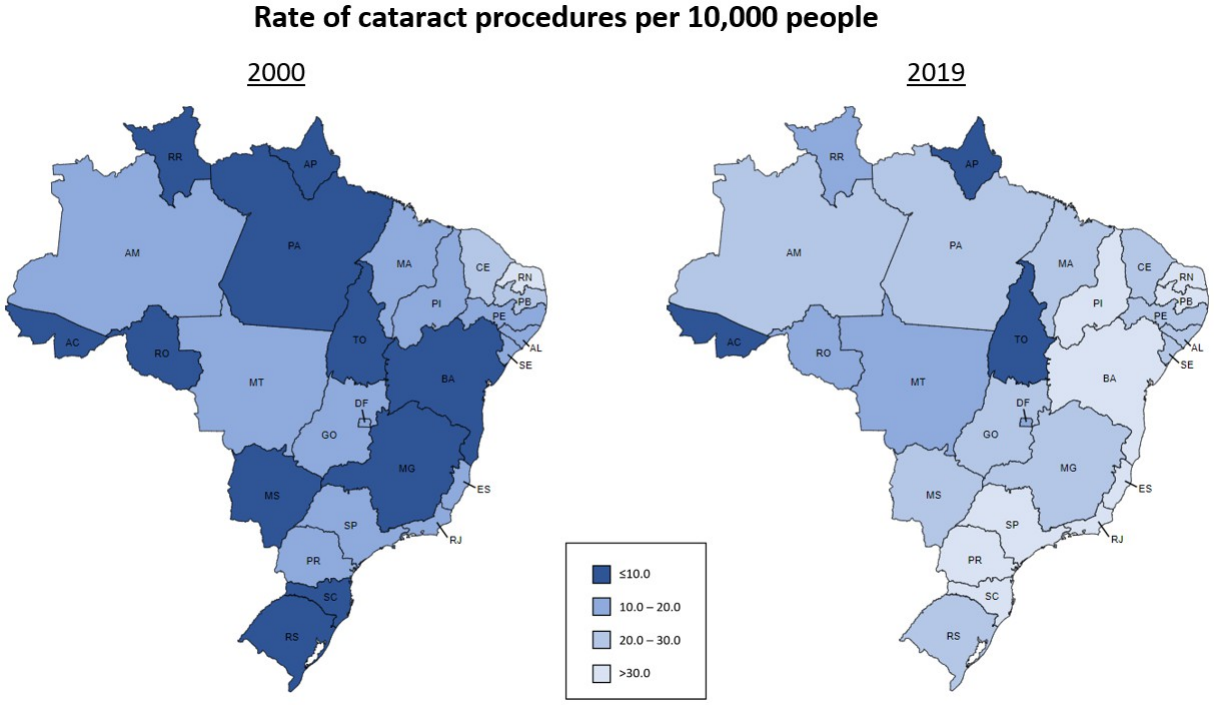
Comparison on rate of cataract procedures per 10,000 people in 2000 and 2019 by state (Source: Go-cart.io [17]).

Fig 2 shows the cataract surgical procedures rate per million people by state in 2000 and 2019. Individual state data per year is available at S1 Table.

The maps indicate an overall improvement on rate of cataract procedures per 10,000 people from 2000 and 2019. Three states from the North Region (AC, AP, and TO), however, showed a persistent low rate along the study period. On the other hand, all the states from regions South (PR, SC, RS), Southeast (MG, ES, RJ, SP) and Northeast (MA, PI, CE, RN, PE, PB, AL, SE, BA) showed rates above 20 procedures per 10,000 people.

**Fig 3:**
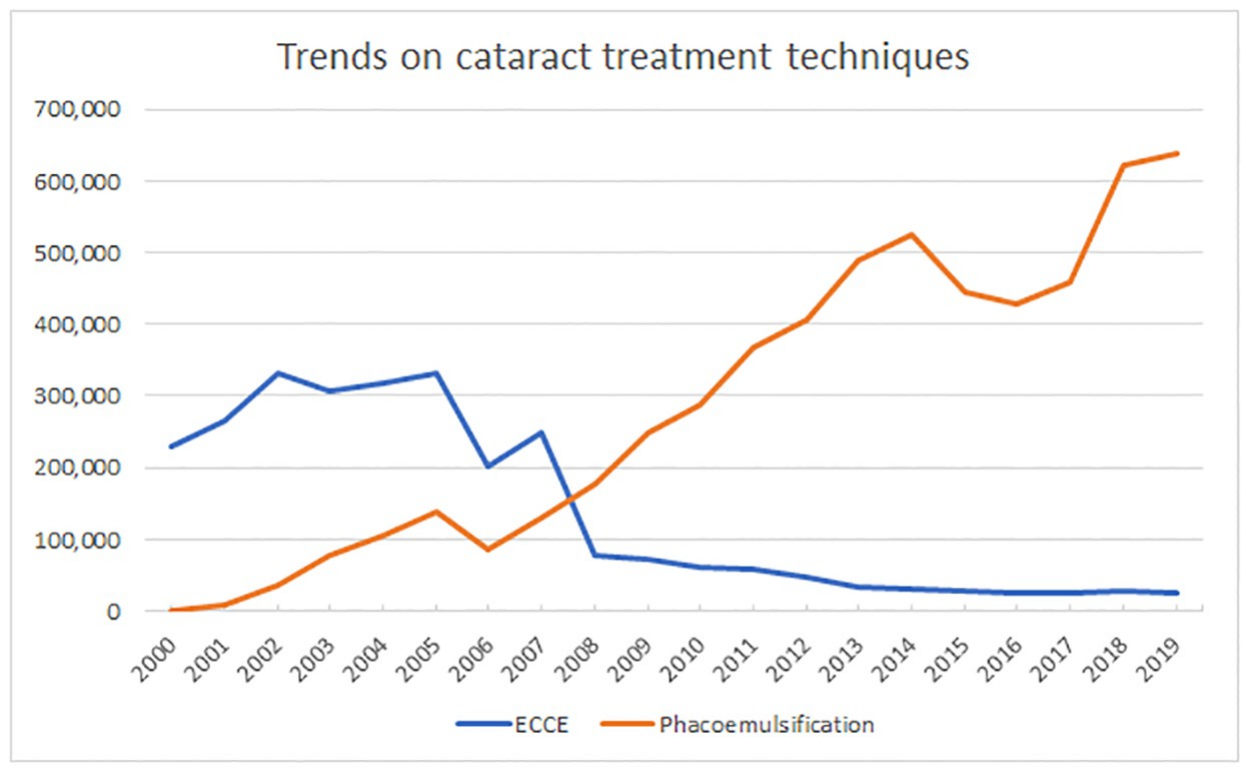
Trends in the absolute number of cataract treatment procedure from 2000 to 2019 according to the technique.

Fig 3 shows the trends on number of procedures according to the technique performed.

It is observed a significant increase on the number of phacoemulsification (Coefficient: 35,446; 95% CI: 31,116 to 39,775; p<0.001) and a significant decrease in the number of ECCE (Coefficient: -18,297; 95% CI: -22,974 to -13,621; p<0.001) along the 20 years period. A shift on the predominant technique has occurred between 2007 and 2008 with phacoemulsification increasing its percentual representativity from 34.3% of all procedures to 69.7%. In 2019, phacoemulsification represented 96.1% of all cataract procedures.

The costs associated to overall cataract treatment in the public system increased from USD$18,109,151.54 in 2000 to USD$101,626,691.25 in 2019, representing an increase of 461.2%. When analyzing each type of treatment separately, the mean cost of phacoemulsification per procedure along the period was USD$126.63, 97.1% higher than the mean cost of ECCE per procedure along the period of USD$64.24. From 2001 to 2019, the phacoemulsification costs per procedure increased 30.5% from USD$119.00 to USD$155.33 (p = 0.007) and the ECCE costs per procedure increased 29.1% from USD$78.57 to USD$101.43 (p = 0.001). Table 3 shows the costs per procedure and total costs for each year. There are no differences in costs per procedure among the regions as costs within the public health system are unique and applied throughout the country.

**Table 3:** Costs in USD of cataract treatment procedures according to year.

Year	Cost per procedure		Total costs in the year
	*Phacoemulsification*	*ECCE*	
**2000**	-	78.57	18,109,151.54
**2001**	119.00	78.57	21,846,159.93
**2002**	119.00	72.05	28,307,401.74
**2003**	119.00	60.84	27,823,262.84
**2004**	119.00	54.06	29,886,030.67
**2005**	119.00	47.39	32,139,187.63
**2006**	119.00	45.53	19,529,300.30
**2007**	119.00	38.24	25,104,858.20
**2008**	83.53	82.24	27,349,241.76
**2009**	91.36	81.80	34,199,662.45
**2010**	97.93	81.76	38,186,119.49
**2011**	102.87	82.01	47,626,062.48
**2012**	107.09	81.56	51,472,214.48
**2013**	111.21	81.41	59,774,196.35
**2014**	113.89	81.78	64,852,197.84
**2015**	114.81	81.68	54,991,449.98
**2016**	114.38	81.36	52,816,026.04
**2017**	122.97	88.01	60,431,760.57
**2018**	152.70	101.43	100,630,623.36
**2019**	155.33	101.43	100,389,704.22

## Discussion

Cataract is indeed one of the main priorities on UEH programs as it is the main cause of visual impairment and blindness worldwide [1, 2]. Recent analysis by ocular public health experts have shown multiple accomplishments along the past 3 decades: the global prevalence of blindness is declining; the contribution of infectious diseases to visual impairment and blindness is declining; and there are more and better data on indicators for blindness prevention (i.e. prevalence and causes of visual impairment and blindness, number of eye care professionals, and cataract surgery service delivery) [8, 9, 18]. On the other hand, as the population gets older and larger, it is expected an increase on the prevalence of visual impairment and blindness due to cataract in the next years; gender and income inequalities are still an issue with previous studies showing women are less likely to get access to cataract surgery and that poverty is associated with reduced access and lower quality of ophthalmic services; despite the growing population of ophthalmologists globally, the professionals are more concentrated in high income countries and in countries with the lowest prevalence of blindness; finally, more data is in need, specially from areas that have not been studied so far [18–21].

In order to evaluate a program progression, national data needs to be analyzed and therefore researchers are urged to develop methods and to generate evidence on the magnitude and causes of visual impairment and eye care services [1, 2]. In that sense, the DATASUS database is a valuable information resource. Data derived from DATASUS has been extensively used for other medical specialties and are important to a better understanding of the health services provided by the Brazilian national health system [16, 22–25]. Some limitations on that strategy, however, might be pointed: the system does not provide data on individual level so that analysis on prevalence of procedures in the population or subgroup analysis according to sex and/or age are limited, and the database brings information only from treatment performed in the public system excluding private health insurance and out-of-pocket procedures.

Our findings show a significant increase on the number of cataract treatment procedures performed in Brazil from 2000 to 2019, following the overall trend of the Latin American subregion [26]. Although the prevalence of cataract blindness has decreased, this is still the leading cause of blindness and vision impairment in all Latin American countries and the need for cataract surgery is likely to increase in the next years [9, 26, 27]. Up to 2005, the Brazilian federal government financed cataract surgeries procedures through Cataract National Campaigns, a national-level program with support of local medical schools aiming to promptly diagnose and treat cataract through SUS. After 2006, however, the program was discontinued with the federal government providing the budget to states, decentralizing the cataract programs. Some states even further decentralized these funds, leading to a municipal management of cataract programs. At such level, the municipalities public health leaders could choose to prioritize other conditions instead of cataract. As a result, we observe a fluctuating, up-down trend of cataract output from 2006 and on [28–30].

When considering one of the UEH core goals, Brazil has integrated the eye health into its wider universal health system, so that in theory, every citizen would have access to eyecare services free of charge. The reality however shows a persistent inequity within the country with substantial differences among the regions in terms of life expectancy, educational level, and gross national income (GNI) which reflects on health care access. To note, the Human Development Index (HDI) in the Northeast, North, South, Midwest, and Southeast regions in 2010 were, respectively, 0.663, 0.667, 0.754, 0.757, and 0.766 [31]. Moreover, the differences in the number of procedures performed in the different regions may also be related to the ophthalmologist’s distribution in the country. According to the WHO, the ideal scenario of ocular health care within a population is a rate of at least 1 ophthalmologist per 17,000 habitants. The most recent census promoted by the Brazilian Council of Ophthalmology in 2019 shows that the country counts with 20,455 ophthalmologists which results in 1 professional per 9,224 population, in accordance to the WHO recommendation. When analyzing each region, however, the rates range from 1 per 7,599 in the Southeast to 1 per 12,084 in the North region [32]. The disparities of concentration of ophthalmologists in the country may impact the frequency of procedures performed in each region. Still, although recent reports have highlighted the importance of considering the effective cataract surgical coverage as a useful measure of progress towards UEH as it evaluates quality, access and, equity, this indicator could not be calculated with the available data, which reinforces the importance on specific data collection schemes [33, 34].

With the available data we calculate rates of procedure per population, which should not be interpreted as cataract surgical rate but can provide an insight of disparities within the country taking into account the differences of population distribution in such a large territory. The results showed that, despite the discrepancies between, some differences are observed even within the different regions. Along the years, most of the regions achieved a consistence in terms of rates of cataract procedure rates, with the region north as the only exception. While states as AM, PA, RR, and RO showed improvement along the 20 years, other as AC, AP, and TO persist with rates below 10 procedures per 10,000 people. Residents from those states rely on punctual cataract surgery campaigns offered in specific years but there is a lack of public health policies for continuous care along the years. This data reinforces the need of individual strategies for each state in order to improve the population access to cataract treatment.

A previous population-based study performed in Sao Paulo in 2004 showed a burden of cataract of 8.54% in adults 50 years and older while a population-based study performed in the Amazon Region in 2014 showed a burden of cataract of 17.23% in adults 45 years and older. Moreover, the surgical coverage was 61.40% in Sao Paulo and 42.12% in the Amazon Region [11, 35]. The higher burden observed in the 2014 may reflect the increasing number of cataract cases along the years. Although a better surgical coverage could be expected as following the increasing number of cataract treatment procedures along the years, it is notable the discrepancies between the regions and, even with a 10-years gap between the studies, the access to cataract surgery was higher in Sao Paulo located in the Southwest region when compared to the Amazon Region located in the North region. As important as seeing the increased number of procedures along the years, it is crucial to guarantee the quality of the provided service. There is a lack of studies analyzing the cataract surgery outcomes in developing countries and these are the main indicator on treatment quality [11, 26, 35]. The two population-based studies performed in Sao Paulo and in the Amazon Region showed frequencies of presenting visual impairment (presenting visual acuity ≥20/63) of, respectively, 30.7% and 34.8% of cataract-operated eyes, significantly above the WHO recommendation of 20% or less [11, 35, 36]. Causes included uncorrected refractive error and/or surgical complications, which highlights the importance of strict treatment protocols and close follow up of patients after surgery in order to improve the service outcomes.

It is clear the shift from ECCE to phacoemulsification technique as the technology spread throughout the country and medical schools adopted it in the surgeons training. In general, surgical outcomes are better in cases of phacoemulsification when in comparison with ECCE, mainly due to lower residual astigmatism and consequently better visual acuity in the early postoperative period [37]. The switch between techniques predominance contributed, therefore, to a more effective decrease on visual impairment and blindness due to cataract in the country. Still, ECCE remains representing a small portion of all procedures as there are cases not indicated to phacoemulsification as eyes with hard nucleus and/or with increased risk of intraoperative complications [11, 38].

The costs associated with the treatment did not follow the same trend observed in the number of procedures, showing non-linear little adjustment of about 30% along the 20 years period. The payment system within SUS adopts a fixed costs table determined by the Ministry of Health for payment of each procedure to the hospitals that perform it, despite of the real costs associated with it. There is a lack of flexibility and absence of periodic adjustments in this table leading to financial challenges on the part of hospitals [39–42]. In order to have a good cost management, it is essential to know how much is spent on each procedure, to accept variations and to perform adequate adjustments, following at least the country’s inflation rates. To notice, according to Brazilian Central Bank, the inflation rate from 2000 to 2019 in Brazil is calculated in 326.23% evidencing the failure on procedures costs adjustment in the period within SUS.

The data evaluated in the current study refers only to the cataract procedures performed through the Brazilian Health System and do not include those performed by nonpublic services as private insurances or under out-of-pocket conditions. Previous studies estimate that two-thirds of cataract surgeries are carried out through SUS, while one-third are performed in private hospitals or private insurance system facilities [29, 30]. No official national data is available as those private services are not required to deposit their data on a unified database as DATASUS.

## Conclusion

There is an increasing trend of procedures related to cataract treatment performed through the Brazilian National Health Insurance along the 20-years period in all regions, in accordance with the Universal Eye Health plan. There was a switch on the treatment technique predominance from ECCE to phacoemulsification, which currently represents the vast majority of procedures. The costs associated with both techniques have not followed the country’s overall inflation. The data derived from DATASUS is important to understand the overall panorama of ocular health offered by the national health system and to provide information to guide healthcare leaders on management and planning of public health policies within the system, however, more detailed data derived from population-based studies are in need to evaluate the national progression towards Universal Eye Health.

## Supporting information

S1 Table Rates of cataract surgical procedures per 10,000 people according to state. (DOCX)
