# Supplementary material for: Trends in cataract surgical treatment within the Brazilian national public health system over a 20-year period: Implications for Universal Eye Health as a global public health goal
Source: PLOS Glob Public Health. 2022 Jun 9;2(6):e0000328. doi: 10.1371/journal.pgph.0000328 (PMC10021674; doi:10.1371/journal.pgph.0000328)
Supplement: S1 Table — (DOCX) [file pgph.0000328.s001.docx]

S1 Table. Rates of cataract surgical procedures per 10,000 people according to state

| **State** | **Region** | **2000** | **2001** | **2002** | **2003** | **2004** | **2005** | **2006** | **2007** | **2008** | **2009** | **2010** | **2011** | **2012** | **2013** | **2014** | **2015** | **2016** | **2017** | **2018** | **2019** |
| --- | --- | --- | --- | --- | --- | --- | --- | --- | --- | --- | --- | --- | --- | --- | --- | --- | --- | --- | --- | --- | --- |
| **RO** | North | 3.46 | 5.24 | 4.90 | 5.48 | 5.27 | 4.82 | 6.99 | 15.30 | 12.16 | 10.49 | 12.11 | 11.54 | 11.04 | 8.96 | 7.26 | 5.23 | 4.86 | 7.59 | 7.86 | 15.28 |
| **AC** | North | 4.17 | 5.27 | 4.54 | 4.96 | 5.58 | 3.16 | 2.87 | 2.79 | 0.44 | 1.40 | 1.10 | 233.62 | 182.09 | 0.80 | 5.90 | 7.06 | 2.00 | 5.48 | 1.51 | 0.78 |
| **AM** | North | 16.17 | 15.41 | 14.00 | 17.57 | 23.97 | 27.82 | 20.71 | 19.35 | 5.88 | 11.28 | 16.92 | 28.01 | 16.51 | 29.20 | 27.18 | 21.02 | 21.95 | 24.45 | 25.79 | 20.88 |
| **RR** | North | 5.91 | 5.95 | 16.59 | 5.83 | 6.68 | 35.26 | 34.10 | 61.92 | 25.20 | 28.78 | 21.69 | 23.17 | 31.14 | 29.69 | 26.22 | 16.04 | 7.90 | 6.43 | 11.79 | 13.43 |
| **PA** | North | 9.69 | 18.47 | 29.28 | 17.80 | 22.13 | 27.07 | 19.08 | 27.15 | 18.90 | 17.12 | 21.26 | 28.41 | 24.63 | 41.76 | 22.17 | 18.79 | 19.45 | 20.54 | 24.43 | 23.56 |
| **AP** | North | 0.12 | 0.13 | 8.13 | 0.68 | 0.35 | 0.18 | 0.39 | 0.21 | 3.22 | 2.69 | 0.00 | 1.64 | 1.32 | 37.76 | 6.21 | 0.00 | 0.00 | 0.00 | 0.00 | 0.43 |
| **TO** | North | 8.10 | 8.93 | 8.43 | 8.99 | 8.14 | 18.10 | 17.97 | 8.48 | 6.89 | 5.79 | 6.35 | 7.72 | 15.72 | 15.86 | 13.31 | 23.29 | 20.24 | 11.88 | 8.67 | 7.09 |
| **MA** | Northeast | 14.20 | 10.81 | 42.06 | 44.83 | 53.61 | 78.45 | 24.42 | 20.58 | 13.63 | 21.00 | 10.86 | 18.97 | 13.37 | 36.23 | 11.27 | 16.73 | 15.67 | 14.11 | 22.64 | 20.08 |
| **PI** | Northeast | 19.61 | 22.87 | 25.82 | 24.29 | 24.02 | 20.79 | 11.88 | 14.06 | 10.54 | 15.07 | 14.29 | 15.56 | 19.62 | 30.28 | 24.66 | 20.44 | 34.87 | 15.07 | 24.68 | 38.58 |
| **CE** | Northeast | 27.04 | 28.93 | 40.59 | 52.00 | 57.16 | 61.86 | 19.42 | 27.72 | 20.22 | 30.30 | 26.85 | 31.32 | 27.98 | 32.81 | 29.51 | 11.94 | 16.38 | 17.64 | 20.95 | 21.09 |
| **RN** | Northeast | 36.70 | 55.54 | 57.78 | 52.18 | 55.20 | 64.35 | 18.79 | 23.86 | 24.22 | 33.62 | 26.71 | 32.13 | 33.88 | 43.17 | 41.08 | 41.92 | 38.38 | 45.25 | 56.48 | 50.19 |
| **PB** | Northeast | 25.97 | 29.66 | 41.60 | 55.35 | 58.46 | 60.63 | 27.82 | 15.96 | 11.96 | 16.57 | 16.59 | 27.56 | 21.11 | 29.79 | 28.44 | 31.79 | 27.84 | 30.62 | 43.01 | 33.18 |
| **PE** | Northeast | 19.41 | 22.61 | 24.61 | 25.10 | 22.11 | 21.73 | 17.29 | 21.57 | 13.01 | 19.27 | 17.07 | 23.26 | 23.08 | 22.33 | 24.03 | 23.20 | 22.64 | 22.17 | 23.26 | 25.57 |
| **AL** | Northeast | 10.17 | 13.24 | 15.82 | 18.24 | 20.91 | 17.48 | 13.86 | 37.11 | 28.55 | 37.91 | 30.20 | 44.00 | 43.55 | 47.72 | 41.07 | 30.50 | 27.69 | 31.69 | 31.09 | 28.71 |
| **SE** | Northeast | 16.62 | 15.03 | 22.29 | 21.09 | 19.68 | 18.47 | 17.31 | 18.73 | 7.33 | 10.33 | 11.48 | 14.15 | 16.09 | 19.09 | 11.16 | 12.55 | 13.70 | 16.30 | 16.94 | 24.41 |
| **BA** | Northeast | 9.68 | 12.24 | 14.75 | 15.32 | 15.26 | 15.58 | 14.44 | 19.22 | 10.43 | 17.56 | 42.26 | 28.20 | 44.97 | 29.67 | 42.09 | 21.29 | 22.70 | 22.11 | 40.82 | 54.19 |
| **MG** | Southeast | 9.19 | 10.15 | 16.03 | 15.01 | 15.76 | 18.70 | 8.98 | 12.30 | 9.74 | 10.66 | 11.73 | 21.60 | 20.34 | 29.91 | 29.94 | 23.13 | 12.34 | 16.11 | 26.64 | 21.19 |
| **ES** | Southeast | 13.91 | 14.56 | 18.74 | 28.60 | 29.06 | 30.56 | 27.43 | 38.76 | 21.22 | 22.65 | 22.83 | 25.86 | 26.73 | 29.71 | 28.40 | 27.72 | 30.44 | 39.78 | 40.63 | 37.80 |
| **RJ** | Southeast | 11.54 | 12.35 | 16.13 | 14.68 | 19.03 | 15.64 | 8.76 | 11.20 | 8.09 | 8.87 | 9.55 | 12.81 | 14.75 | 15.87 | 19.12 | 17.81 | 14.68 | 13.48 | 28.38 | 31.33 |
| **SP** | Southeast | 13.38 | 16.44 | 20.85 | 20.70 | 22.60 | 24.23 | 16.51 | 23.65 | 14.85 | 16.43 | 17.36 | 19.45 | 21.26 | 25.87 | 27.11 | 26.54 | 26.09 | 29.31 | 33.96 | 35.77 |
| **PR** | South | 12.92 | 12.31 | 14.88 | 14.57 | 16.59 | 18.07 | 13.58 | 19.07 | 12.32 | 17.64 | 13.14 | 15.79 | 18.25 | 21.39 | 27.03 | 26.26 | 29.89 | 30.83 | 38.13 | 36.80 |
| **SC** | South | 6.16 | 6.12 | 9.08 | 12.39 | 13.09 | 13.16 | 13.66 | 17.98 | 7.84 | 13.80 | 12.58 | 15.69 | 16.34 | 18.46 | 23.56 | 23.58 | 16.89 | 21.64 | 53.23 | 63.40 |
| **RS** | South | 9.08 | 10.15 | 11.45 | 13.52 | 13.39 | 17.11 | 19.00 | 21.03 | 13.47 | 14.16 | 15.98 | 14.46 | 16.28 | 18.89 | 20.94 | 23.93 | 21.15 | 24.02 | 24.50 | 28.22 |
| **MS** | Midwest | 6.13 | 13.09 | 16.24 | 19.80 | 22.84 | 20.08 | 21.19 | 28.63 | 16.21 | 16.51 | 15.91 | 19.35 | 21.78 | 17.98 | 15.17 | 65.44 | 97.01 | 16.71 | 42.09 | 28.46 |
| **MT** | Midwest | 10.91 | 16.61 | 19.13 | 14.27 | 10.64 | 9.74 | 3.54 | 10.21 | 11.36 | 18.47 | 16.49 | 18.29 | 20.78 | 17.37 | 14.95 | 13.06 | 14.78 | 59.83 | 57.13 | 11.98 |
| **GO** | Midwest | 10.59 | 18.75 | 22.50 | 18.28 | 22.96 | 20.81 | 15.00 | 15.78 | 19.42 | 23.49 | 18.35 | 20.32 | 21.09 | 24.49 | 27.99 | 24.91 | 19.06 | 23.67 | 27.01 | 23.22 |
| **DF** | Midwest | 15.44 | 17.41 | 10.60 | 22.81 | 29.19 | 32.41 | 12.43 | 24.51 | 13.38 | 5.28 | 4.98 | 8.22 | 8.21 | 6.72 | 120.52 | 3.69 | 6.16 | 5.61 | 5.79 | 10.04 |
| **All** |  | 13.15 | 15.59 | 20.68 | 21.25 | 23.23 | 25.36 | 15.40 | 20.08 | 17.16 | 19.73 | 20.46 | 24.05 | 24.70 | 27.18 | 28.52 | 23.80 | 22.75 | 24.01 | 31.93 | 32.28 |
